# Spontaneous and cued gaze-following in autism and Williams syndrome

**DOI:** 10.1186/1866-1955-5-13

**Published:** 2013-05-10

**Authors:** Deborah M Riby, Peter JB Hancock, Nicola Jones, Mary Hanley

**Affiliations:** 1 School of Psychology, Newcastle University, Newcastle upon Tyne, NE1 7RU, UK; 2 Psychology, School of Natural Sciences, University of Stirling, Stirling, FK9 4LA, UK; 3 Department of Psychology, Northumbria University, Newcastle upon Tyne, NE1 8ST, UK; 4 School of Psychology, Queen's University Belfast, Belfast, BT7 1NN, UK

**Keywords:** Williams syndrome, Autism, Gaze behavior, Social attention, Social cognition

## Abstract

**Background:**

From a young age the typical development of social functioning relies upon the allocation of attention to socially relevant information, which in turn allows experience at processing such information and thus enhances social cognition. As such, research has attempted to identify the developmental processes that are derailed in some neuro-developmental disorders that impact upon social functioning. Williams syndrome (WS) and autism are disorders of development that are characterized by atypical yet divergent social phenotypes and atypicalities of attention to people.

**Methods:**

We used eye tracking to explore how individuals with WS and autism attended to, and subsequently interpreted, an actor’s eye gaze cue within a social scene. Images were presented for 3 seconds, initially with an instruction simply to look at the picture. The images were then shown again, with the participant asked to identify the object being looked at. Allocation of eye gaze in each condition was analyzed by analysis of variance and accuracy of identification was compared with *t* tests.

**Results:**

Participants with WS allocated more gaze time to face and eyes than their matched controls, both with and without being asked to identify the item being looked at; while participants with autism spent less time on face and eyes in both conditions. When cued to follow gaze, participants with WS increased gaze to the correct targets; those with autism looked more at the face and eyes but did not increase gaze to the correct targets, while continuing to look much more than their controls at implausible targets. Both groups identified fewer objects than their controls.

**Conclusions:**

The atypicalities found are likely to be entwined with the deficits shown in interpreting social cognitive cues from the images. WS and autism are characterized by atypicalities of social attention that impact upon socio-cognitive expertise, but, importantly, the type of atypicality is syndrome specific.

## Background

A variety of face skills are critical to social communication; for example, interpreting expressions of emotion or identifying people we know from strangers. The current work focuses specifically on the interpretation of eye gaze cues. Eye gaze plays a central role in communication; for example, signaling turn-taking during conversations [1]. For typically developing (TD) adults, shifts of eye gaze trigger a reflexive orienting of attention [2] in an attempt to align and share interests between individuals [3]. Further down the developmental spectrum, newborn infants can differentiate the basic direction of gaze cues (direct versus averted [4]) and from 3 months old can follow an adult’s gaze shift [5]. A sophisticated understanding of gaze (the mentalistic representation) is likely to show more protracted development with the emergence of theory of mind ability [6]. For some individuals who are developing atypically, interpreting gaze cues may be especially difficult. This is likely to be the case for individuals with autism and, although less prominently researched, for individuals with Williams syndrome (WS). Importantly, the atypical orientation of gaze to social information throughout development may impact upon more sophisticated socio-cognitive understanding.

The neuro-developmental disorders WS and autism are characterized by atypical social interaction styles that have implications for socio-communicative functioning. However, the precise nature of these atypicalities provides clinical insights into opposing behavioral phenotypes. In early infancy, both WS (estimated prevalence ranges from 1:7,500 to 1:20,000 [7,8]) and autism (estimated prevalence for ‘typical’ autism, 7.1:10,000 [9]) are characterized by atypicalities in the use of gesture, pointing and joint attention but the atypical development of these and other socially relevant skills results in divergent behaviors when interacting with other people [10-12]. Individuals with autism spectrum disorders are typically characterized by social withdrawal and isolation [13], whereas those with WS are reported to show hyper-sociability or a pro-social drive [14,15]. Perhaps the most important social cue to be deciphered for interpersonal communication is the human face. Previous research involving autism and WS has emphasized atypicalities in the way that faces are attended to [16,17] and subsequently processed [18,19]. The current research explores attention to face information as an exploration of social attention orientation and subsequent socio-cognitive processing.

To interpret information from faces we must first attend to them. A well-recognized fact is that individuals with autism fail to orient to socially salient information that would typically capture attention [20]. Not only does socially relevant information (for example, faces) fail to capture attention in a typical manner, once faces are detected the distribution of attention throughout facial regions occurs atypically. When attending to static faces, individuals with autism show reduced fixation to the highly salient eye region [16,21,22]. In contrast, individuals with WS show prolonged facial attention, especially towards the eye region [16,17], and may rely upon use of the eyes more than is typical for various face tasks – for example, identity matching [23] and mental state recognition [24]. An atypical allocation of attention to faces throughout development will have consequences for detecting the range of subtle face cues that are central to social communication for individuals with both autism and WS.

Eye tracking has been used to explore various aspects of attention to faces. In research with adults who have developed typically, Castelhano and colleagues explored the importance of an actor’s gaze cues for guiding attention in social scene pictures [25]. The item being attended to by the actor was fixated more than any other region of the picture. One could conclude that the adults were able to realize the social importance of the actor’s gaze and thus allocate their own attention accordingly (perhaps then following this with a socio-cognitive judgment about the actor and their desires). In similar work, the same authors found following a face fixation that a typical adult participant was most likely to directly fixate upon the target of the actor’s gaze, compared with a different object [26]. Typical adult viewers appear extremely sensitive to an actor’s gaze direction, which can be used to guide their own attention and subsequently make judgments about the information they are attending to.

The attentional response to eye gaze cues in autism has been broadly studied and found to be atypical across various paradigms – for example, Posner-type cueing [27] and response to joint attention [28]. However, the evidence regarding the selection of eye gaze cues and spontaneous gaze-following by individuals on the autism spectrum is less consistent. For example, while it has been reported that high-functioning individuals with autism (without additional learning difficulties and thus with an IQ within the normal range) showed a reduced likelihood to spontaneously follow an actor’s gaze within a social picture [29], other research has reported seemingly typical gaze cueing [30]. Importantly, both studies included high-functioning participants with autism and therefore any difference between studies seems unrelated to the level of functioning. No such research has to date explored this issue in individuals with WS. The question of whether individuals with WS and autism can follow gaze is an important one because the atypical allocation of attention when perceiving socially relevant information will have a subsequent effect on the appropriate interpretation of that information, relating to the more cognitive aspects of social information processing.

### Using eye tracking to explore components of cognitive performance

There has been a recent surge in research using eye tracking with individuals who have disorders of development *during* task completion to unearth possibly atypical processing strategies. Research involving individuals on the autism spectrum has used eye tracking to explore emotion recognition ability [31-33], the effect of face-familiarity on face perception [34] and eye direction detection within a basic gaze-cueing paradigm [35]. More widely the method has been applied to language processing [36], communicative competence [37], imitation skill [38] and visual search strategies [39] of individuals with autism. Other research has been applied to other populations such as attention-deficit hyperactivity disorder, schizophrenia [40] and WS [16,17,41,42]. Together these studies emphasize that eye tracking can be valuable in unearthing strategies that underlie task performance [43], and indeed identifying atypicalities of attention allocation may allow us to infer the timing of any breakdown in subsequent cognitive processing.

### Current aims

The current study will explore aspects of gaze behavior in WS and autism. Including the two populations together will allow us to consider relevant aspects of their atypical and divergent behavioral and cognitive phenotypes [11,12]. Participants will attend to pictures under two conditions: uncued (spontaneous viewing), and cued to detect the target of an actor’s gaze (thus requiring the participant to use socio-cognitive interpretation skills). The study will therefore explore any atypicalities of the allocation of attention during social perception and follow this by exploring any atypical interpretations of social information at a cognitive level. Based on previous research we derive a number of specific hypotheses. First, in both WS and autism there will be evidence for atypical allocation of attention to social information (in both conditions), thus suggesting atypical social perception in both groups. Explicitly, we predict that individuals with autism will attend to faces for a shorter time than is typical [16,20] and individuals with WS will show prolonged face fixation [16]. Second, individuals with both WS and autism will show socio-cognitive deficits that are evident by their poor ability to follow gaze and identify the target item that the actor is looking at in the scene. Explicitly, lower accuracy will be evident for the WS and autism groups (compared with TD individuals) when naming the target object that the actor is looking at.

## Methods

### Participants

Eighteen participants with WS were recruited via the Williams Syndrome Foundation to participate in eye tracking tasks reported here and elsewhere [16,17,41]. All participants had been diagnosed clinically and had previously had their diagnosis confirmed with genetic fluorescent *in situ* hybridization testing to detect the deletion of one copy of the elastin gene on chromosome 7 (7q11.23 [44]). All participants with WS had normal or corrected-to-normal vision and none had strabismus. Three individuals were removed due to recording/task compliance difficulties. The final sample consisted of 15 WS participants between 8 years 8 months and 28 years 0 months old (mean, 13 years 6 months; 11 male, four female).

Each WS participant was individually matched to a typically developing (TD) individual of comparable nonverbal ability. The decision to match groups on nonverbal ability relates to the nonverbal nature of the spontaneous attention allocation phase of the study and also the gaze cue provided by the actor. Having previously involved all participants in eye-tracking research, the participants were all familiar with eye-tracking procedures. All participants had normal or corrected-to-normal vision. TD participants were recruited from local schools. Teachers completed the Strengths & Difficulties Questionnaire [45], reporting behavior within the normal range. The Strengths & Difficulties Questionnaire is a 25-item questionnaire that provides measures of ‘emotional symptoms’, ‘conduct problems’, ‘hyperactivity’, ‘peer problems’, and ‘prosocial behaviour’. A ‘total difficulties’ score can be calculated for each individual, and to score within the ‘normal range’ implies that the individual shows no atypicality of behaviors that impact upon their everyday life. The TD and WS groups were matched using the Ravens Coloured Progressive Matrices task (maximum score 36) [46]. The WS group scored between 9 and 21 (mean 15) and the TD group scored between 9 and 23 (mean 15, difference *P* = 0.74). The TD group was significantly younger than the WS group (mean age, 10 years 1 month; *t*(28) = 4.94, *P* <0.001).

Twenty-six child and adolescent participants with autism were recruited via mainstream schools/specialized education units and all had normal or corrected-to-normal vision. Participants had previously been clinically diagnosed according to the Diagnostic and Statistical Manual of Mental Disorders, Fourth Edition [47]. The Childhood Autism Rating Scale (CARS) was completed by teachers [48] and classified 15 children as mild-moderately autistic and 11 as severely autistic (scores ranged between 33 and 41). This measure has previously been reported to correlate level of functioning with attention to faces [17]. Due to task compliance and/or calibration difficulties, four participants were removed. The final sample consisted of 22 individuals aged 7 years 11 months to 17 years 6 months (mean, 11 years 3 months; 18 male, four female; CARS score, 33 to 40). Participants with autism were matched to a TD individual of comparable nonverbal ability, who all scored within the normal range on the Strengths & Difficulties Questionnaire. On the Ravens Coloured Progressive Matrices, the autism group scored between 8 and 19 (mean 12) and the TD group scored between 7 and 18 (mean 13, difference *P* = 0.70). The TD group was significantly younger than the autism group (mean age, 9 years 2 months; *t*(21) = 2.59, *P* <0.05). See Table 1 for a summary of important participant characteristics.

**Table 1:** Key participant characteristics across groups

	**Final number**	**Number excluded**	**Chronological age**^**a**^	**RCPM scores**^**b**^	**SDQ scores**^**b**^	**CARS scores**^**b**^
Williams syndrome	15	3	13 years 6 months (70 months)	15 (5.0)	N/A	N/A
TD matches	15	0	10 years 1 month (49 months)	15 (5.0)	7 (2)	N/A
Autism	22	4	11 years 3 months (62 months)	12 (3.7)	N/A	39 (4)
TD matches	22	0	9 years 2 months (51 months)	13 (3.5)	8 (2)	N/A

Standard deviation presented in parentheses. ^a^Standard deviation in full calendar months.

^b^Group mean scores for the Ravens Coloured Progressive Matrices (RCPM), the Strengths & Difficulties Questionnaire (SDQ) and the Childhood Autism Rating Scale (CARS) are rounded to the nearest whole number and therefore the nearest possible score on these measures.

The neuro-developmental disorder groups (WS, autism) and their TD matches were not matched on gender because there is a lack of empirical evidence to suggest a theoretical link between gender and the allocation of attention. There is also equivocal evidence concerning the role of gender in gaze-cueing effects [49,50]. Similarly, we did not match groups based on chronological age because it was highly unlikely that individuals with the neuro-developmental disorders would perform at age-appropriate levels in the socio-cognitive cued condition and because previous research has indicated no significant correlation between chronological age and attention allocation to faces in WS, autism or TD [17].

Informed consent and favorable ethical approval were received prior to the study from the research ethics committee in the Department of Psychology at Stirling University.

### Materials and design

**Figure 1:**
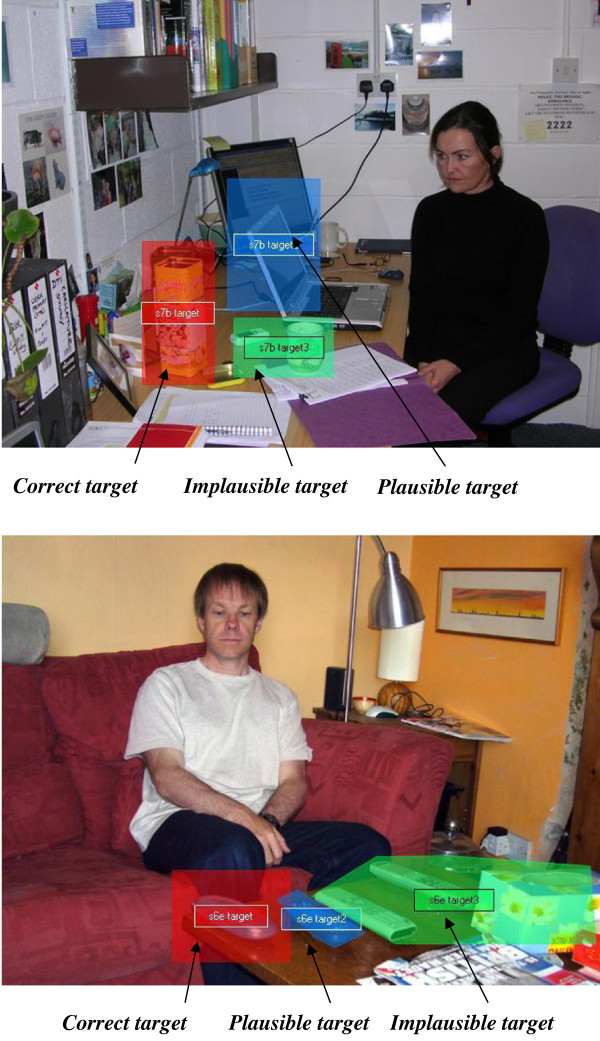
**Examples of two scenes used in the study with the target items highlighted.** All images were shown in full color during the experiment and are available in Additional file 1. Those portrayed in these images are volunteers who consented to the use of their images in the study, not participants.

Color digital photographs were taken using a Nikon CoolPix 4100 camera (Nikon UK Ltd, Kingston upon Thames, UK). All images were standardized for size (640×480 pixels) using Adobe Photoshop, giving a visual angle of 20×15° at the viewing distance of 60 cm. Actors appeared in different settings across images, with one actor in each picture, and the actor’s gaze was directed to a target item in the complex scene. The actors were adults who were unfamiliar to the participants. The setting varied across stimuli; for example, an office, kitchen and lounge (see Figure 1 and Additional file 1). There were 14 different images involving seven actors (three male, four female). The target item differed across images (for example, remote control, glasses case, a mug, a pen) and appeared alongside various naturally occurring distracter items. The location of the actor and target item varied across images. Participants viewed each scene for 3 seconds in a random order. A set time of 3 seconds was used to prevent exhaustive scanning of the images. There was a 1 second inter-stimulus blank screen.

Gaze behavior was recorded via a portable Tobii 1750 eye-tracker run using TobiiStudio (Tobii Technology AB, Danderyd, Sweden). The eye tracker was interfaced and controlled via a Dell Latitude D820 laptop (Dell Corporation Ltd, Bracknell, UK). The eye-tracking system was completely non-invasive with no requirement to constrain head movements. The system tracked both eyes, to a rated accuracy of 0.5°, sampled at 50 Hz. It was calibrated for each participant using a 9-point calibration.

Areas of interest (AOIs) were designated to each scene. AOIs were assigned to: the whole scene; the face region, following the face outline; the actor’s eye region, drawing a rectangular shape to encompass the eyes; and target items. Three classes of target were identified by the authors: the correct target was the item actually being looked at; other objects judged to be potentially in the line of sight were labeled as plausible targets; while other objects not in the line of sight, either in the wrong direction or in the right direction but clearly behind the observer, were labeled as implausible targets. Examples of the three target types are shown in Figure 1, while the complete set is presented in Additional file 1. The TobiiStudio package exported gaze fixation duration (milliseconds) to each AOI across scenes for each participant. We also recorded the time to first fixation on each AOI; these data are reported in Additional file 2.

### Procedure

Participants were tested in a quiet setting either in their school or in their home and they sat approximately 50 cm from the screen with the experimenter beside them. The eye tracker was calibrated and if this process failed the participant was removed from the study. The participant always completed the spontaneous allocation of attention phase (uncued) prior to the socio-cognitive phase (cued phase; to avoid cueing affecting spontaneous attention allocation). Critically, all participants completed both conditions and all trials in each condition. In the uncued spontaneous allocation condition, participants were instructed to ‘look at each picture for as long as it remains on screen’ (3 seconds). In the cued social cognition condition, they were told to ‘detect and name what the actor is looking at’ (the experimenter recorded the verbal response). In this condition the stimulus still remained on screen for 3 seconds even if the participant gave their response before this time expired. At the end of the experiment we ensured that the participant was able to identify all of the different target items that had been used in the task.

## Results

The eye-tracker data indicated that all participant groups were attending to the displayed images on average for more than 90% of the time that they were on screen, with the exception of the TD matches to the WS group in the cued condition who averaged 84%, presumably because they were disengaging from the screen once they had answered the question. Formal comparison of this measure of task engagement revealed no significant difference between the groups (*P* >0.05). Nevertheless, we computed gaze to AOIs as a proportion of the total engagement time for each individual to remove this source of individual variation.

**Figure 2:**
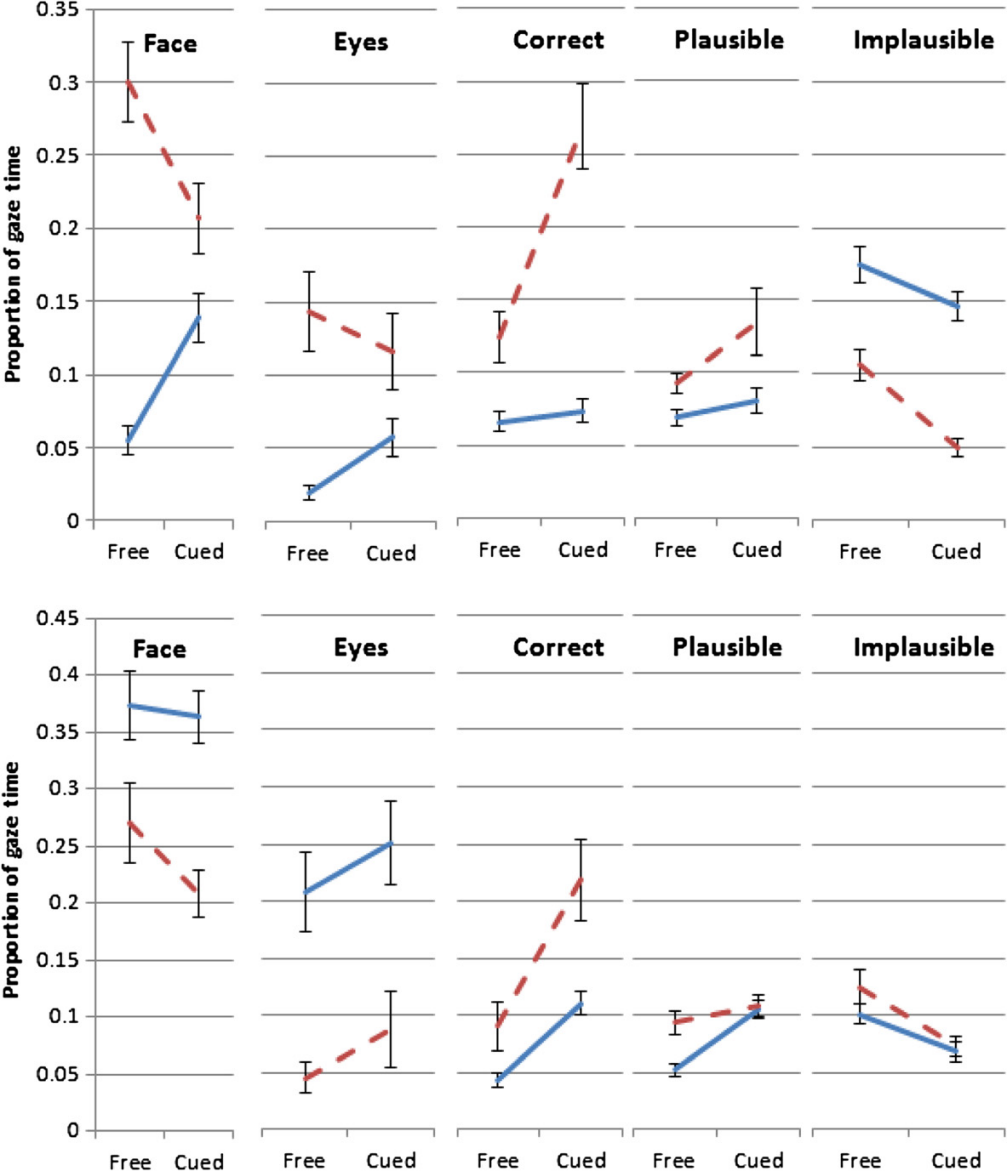
**Proportions of gaze time to each area of interest for free (spontaneous) and cued gaze.** Top panel: autism group (solid lines) and their typical control group (dashed line). Bottom panel: Williams syndrome group (solid lines) and their typical control group (dashed line).

Gaze behaviors of participants in the WS and autism groups were compared with their respective TD comparison group. The AOIs (face, eyes, correct, plausible and implausible targets) were used for analysis. The AOI for the face includes the eye region. In the interest of clarity we report only results that are both significant and relevant: for example, it is uninformative that there is a strong main effect of AOI on gaze behavior throughout. Figure 2 shows the overall pattern of results for both gaze conditions and all participant groups. The figure specifically indicates the effect of task instruction on gaze allocation to the different AOIs per group. Movie files illustrating the time course of cued gaze patterns are shown in Additional files 3, 4 and 5.

### Autism

Participants with autism were compared to their TD matches using a 2×2×5 analysis of variance (ANOVA) with the independent factor Group (Autism, TD) and repeated factors of Condition (spontaneous, cued) and AOI (face, eyes, correct, plausible and implausible targets). There was a significant three-way interaction, *F*(4,168) = 11.6, *P* <0.001, *ŋ*^2^_p_ = 0.22. To understand the source of this interaction we ran 2×5 ANOVAs to compare the two groups within each viewing condition and the two viewing conditions within each group.

#### Comparison across viewing condition, within participant group

Separate 2×5 ANOVAs showed an interaction between AOI and Condition for both participants with autism, *F*(4,84) = 11.9, *P* <0.001, *ŋ*^2^_p_ = 0.36, and their TD matches, *F*(4,84) = 8.72, *P* <0.001, *ŋ*^2^_p_ = 0.29. There was a significant effect of Condition for those with autism, *F*(1,84) = 9.84, *P* = 0.005, *ŋ*^2^_p_ = 0.32 (mean gaze to AOIs in spontaneous viewing = 0.077, when cued = 0.099) but not for the TD matches, *F*(1,84) = 0.07. Only the participants with autism increased their average gaze across the labeled AOIs in the cued compared with uncued viewing condition.

**Figure 3:**
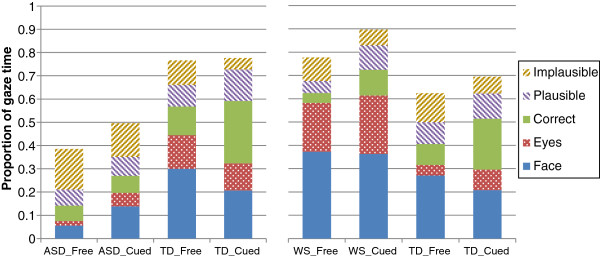
**Gaze to areas of interest for free (spontaneous) and cued gaze, and each participant group.** The proportions do not stack to 100% because of time spent looking at areas of the images outside the areas of interest (AOIs). Left panel: participants with autism (ASD) and their typically developing (TD) matches. Right panel: participants with Williams syndrome (WS) and their TD matches.

To interpret the AOI by Condition interactions, paired *t* tests were run to compare gaze time to each AOI in each viewing condition (see Figure 3). Participants with autism spent significantly longer looking at the face, *t*(21) = 4.34, *P* <0.001, and at the eyes, *t*(21) = 3.41, *P* = 0.003, in the cued condition, and marginally less time, *t*(21) = 1.98, *P* = 0.061, looking at the implausible targets. Time spent fixating on the correct and plausible targets did not differ, both *P* >0.1. The TD matches spent less time looking at the face, *t*(21) = 2.68, *P* = 0.014, and at the implausible targets, *t*(21) = 4.45, *P* <0.001, and more time looking at the correct target, *t*(21) = 4.19, *P* <0.001, in the cued condition. Time on the eyes and the plausible targets did not differ, both *P* >0.1. In summary, the TD group shifted their attention from face to correct target in the cued condition, while those with autism shifted their attention towards the face but did not show a transfer to correct target.

#### Comparison between participant groups, within viewing condition

Separate 2×5 ANOVAs showed an interaction between AOI and participant group in both spontaneous viewing, *F*(4,168) = 29.5, *P* <0.001, *ŋ*^2^_p_ = 0.41, and when cued, *F*(4,168) = 16.7, *P* <0.001, *ŋ*^2^_p_ = 0.29. There was a significant effect of participant group in both the spontaneous viewing condition, *F*(1,42) = 63.6, *P* <0.001, *ŋ*^2^_p_ = 0.60, and when cued, *F*(1,42) = 19.7, *P* <0.001, *ŋ*^2^_p_ = 0.32. In both conditions, the TD matches spent longer on the AOIs than those with autism (see Figure 3).

To interpret the interactions, independent *t* tests compared viewing time for each participant group to each AOI. During spontaneous viewing, the TD group spent longer than those with autism looking at the face, *t*(26.5) = 8.47, *P* <0.001, at the eyes, *t*(22.2) = 4.44, *P* <0.001, at the correct targets, *t*(26.7) = 3.11, *P* = 0.004, and at the plausible targets, *t*(42) = 2.51, *P* = 0.016, but less time looking at the implausible targets, *t*(26.7) = 4.17, *P* <0.001. A similar pattern held when viewing was cued, with the TD group spending longer on the face, *t*(42) = 2.31, *P* = 0.026, on the correct targets, *t*(24.3) = 6.43, *P* <0.001, and on the plausible targets, *t*(42) = 2.23, *P* = 0.031, marginally longer on the eyes, *t*(42) = 2.01, *P* = 0.051, and much less time looking at the implausible targets, *t*(42) = 8.29, *P* <0.001. In summary, the TD group spent more time on the face and, perhaps by natural gaze-following, on the correct and plausible targets, while those with autism spent more time looking around the whole image and at implausible targets, even in the cued viewing condition.

A correlation between CARS score for the Autism group (as an indication of level of functioning on the autism spectrum) and fixation length to the target item was not significant either for spontaneous viewing (*r* = −0.3, *P* = 0.18) or for cued viewing (*r* = −0.04, *P* = 0.85).

#### Behavioral performance

Participants with autism were significantly less accurate than their typical matches at naming the target item in the cued condition *t*(42) = 4.18, *P* <0.001 (mean autism, 7 items, SD 2.6; mean TD, 10 items, SD 1.5). The correlation between CARS score and behavioral performance indicated that individuals who were higher functioning scored more accurately (*r* = −0.49, *P* <0.05).

### Williams syndrome

Participants with WS were likewise compared with their TD matches using a 2×2×5 ANOVA with an independent factor of Group and repeated factors of Condition and AOI. The three-way interaction was not significant, *F*(4,112) = 1.40, *P* = 0.24; however, there were significant interactions between gaze condition and AOI, *F*(4,112) = 9.99, *P* <0.001, *ŋ*^2^_p_ = 0.26, and between participant group and AOI, *F*(4,112) = 18.3, *P* <0.001, *ŋ*^2^_p_ = 0.40. The interaction between gaze condition and participant group was not significant, *P* = 0.44. To understand the source of the two interactions we again ran 2×5 ANOVAs to compare the two groups within each viewing condition and the two viewing conditions within each group.

#### Comparison across viewing condition, within participant group

Separate 2×5 ANOVAs showed an interaction between AOI and viewing condition for both participants with WS, *F*(4,56) = 2.89, *P* = 0.03, *ŋ*^2^_p_ = 0.17, and for their TD matches, *F*(4,56) = 8.13, *P* <0.001, *ŋ*^2^_p_ = 0.37. There was a significant effect of Condition for those with WS, *F*(4,56) = 11.3, *P* = 0.005, *ŋ*^2^_p_ = 0.45 (mean gaze to AOIs in spontaneous viewing = 0.16, when cued = 0.18) but not for the TD matches, *F*(1,84) = 1.05, *P* = 0.17. Participants with WS also increased their average gaze across the labeled AOIs in the cued viewing condition.

To interpret the AOI by viewing condition interactions, paired *t* tests were run to compare gaze time to each AOI in each Condition. Participants with WS did not significantly change their gaze to face or eyes (both *P* >0.1) but spent significantly longer looking at the correct target *t*(14) = 6.60, *P* <0.001, and the plausible targets, *t*(14) = 6.08, *P* <0.001, in the cued gaze condition. They spent less time looking at the implausible targets, *t*(14) = 3.74, *P* = 0.002. The TD matches showed a similar pattern, with no significant change to eyes or face (*P* >0.1), more time looking at the correct targets, *t*(14) = 3.87, *P* = 0.002, less looking at the implausible targets, *t*(14) = 3.50, *P* = 0.004, but no change to the plausible targets, *P =* 0.27. In summary, the participants with WS and their TD counterparts showed a similar pattern, shifting gaze towards the correct targets and away from implausible ones when cued to follow gaze.

#### Comparison between participant groups, within viewing condition

Separate 2×5 ANOVAs showed an interaction between AOI and participant group in both spontaneous viewing, *F*(4,112) = 10.3, *P* <0.001, *ŋ*^2^_p_ = 0.26, and when cued, *F*(4,112) = 13.7, *P* <0.001, *ŋ*^2^_p_ = 0.33. There was a significant effect of participant group in both the spontaneous viewing condition, *F*(1,28) = 5.47, *P* = 0.027, *ŋ*^2^_p_ = 0.16, and when cued, *F*(1,28) = 7.82, *P* = 0.009, *ŋ*^2^_p_ = 0.22. In both conditions, those with WS spent longer on the AOIs than the TD matches (in marked contrast to those with autism).

To interpret the interactions, independent *t* tests compared viewing time for each participant group to each AOI. During spontaneous viewing, the WS group spent longer than the TD matches looking at the face, *t*(28) = 2.24, *P* = 0.033, and at the eyes, *t*(18.5) = 4.34, *P* <0.001, but less time at the correct targets, *t*(28) = 2.14, *P* = 0.041, and at the plausible targets, *t*(28) = 3.45, *P* = 0.002; there was no difference to implausible targets, *P* = 0.18. Again, a similar pattern held when viewing was cued, with the WS group spending longer on the face, *t*(28) = 5.05, *P* <0.001, and eyes, *t*(28) = 3.35, *P* = 0.002 but less on the correct targets, *t*(16.5) = 2.94, *P* = 0.009. There was no difference in gaze allocated to the plausible and implausible targets, both *P* >0.6. In summary, the WS group showed more gaze to the face and less shifting to the target, both during spontaneous viewing and when cued to follow gaze.

#### Behavioral performance

Participants were given a score of 1 per trial for correctly identifying target items and 0 for incorrectly identifying items in the cued condition (maximum 14). Participants with WS were significantly less accurate that their typical matches at making the socio-cognitive judgment and naming the target item, *t*(28) = 2.16, *P* <0.05 (mean WS, 9 items, SD 1.7; mean TD, 11 items, SD 1.4).

#### Typically developing groups

Figure 2 suggests a surprising difference between the gaze behavior of the two TD groups, especially in the spontaneous condition: the TD matches for the autism group appear to spend a greater proportion of the time looking at the eyes than do the TD matches for the WS group. The two control groups are not really comparable, since they are not equivalent, but to check the apparent oddity a 5 (AOI)×2 (TD group) mixed ANOVA was run and showed that the interaction was not significant, *F*(4,140) = 2.18, *P* = 0.074. Note also that this apparent difference is not evident in the time to first fixation, available in Additional file 2.

## Discussion

The current findings support suggestions of both the atypical allocation of social attention in WS and autism and problematic eye gaze interpretation linked to deficits of social cognition in these groups. The atypical allocation of attention seen here is syndrome specific and mirrors that previously reported in the literature on attention to faces [16]. From the current study we can therefore further propose syndrome-specific signatures of atypical attention allocation in WS and autism. For example, individuals with WS over-attend to faces compared with TD individuals while those with autism under-attend to the same information [16]. The difference between the developmental disorders is further highlighted by the patterns of gaze observed when asked to decide what the person shown is looking at. The current study makes a significant new contribution by adding consideration of attention to plausible and implausible incorrect items within the scene images. In this specific case, plausibility is defined only by the gaze of the actor – but it may also be associated with other factors in everyday settings, such as gender and gender-specific targets or age. In the current data, participants with WS resemble their TD matches, increasing gaze to both the correct and plausible targets and decreasing it to implausible ones when attention is cued. The difference is that the individuals with WS remain much more engaged with the face and eyes and are somewhat less successful in identifying the correct target than those developing typically. Those with autism evidently understand that, to answer the question, they need to look more at the actor’s face and eyes but then show little evidence in the fixation data of successfully following the actor’s gaze and continue to look at implausible areas of the image.

One can propose that both over-attending and under-attending to social information (in this case faces) is problematic for the typical development of social cognition. Over-attending in WS is thus as deficient as under-attending in autism. Furthermore, observable similarities at the behavioral level may be associated with very different underlying atypicalities in these groups, as revealed by the current use of eye-tracking methodology. Critically, problems interpreting subtle facial signals will have implications for inter-personal communication in both populations.

The link between attending to faces and a sophisticated understanding of facial cues requires further exploration, especially when interpreting the results of individuals with WS. Purely attending to faces versus the sophisticated interpretation of face cues is very different. The face gaze of individuals with WS was atypical in both conditions assessed here, but atypically prolonged attention to faces did not allow for, or provide, adequate interpretation of gaze cues (certainly in the time that was available to them and using this one parameter of gaze behavior: fixation length). There is probably a very complex relationship between attention and more cognitive interpretation, which may be different in typical and atypical development, and indeed different between different syndromes. Indeed other aspects of gaze behavior (such as time to fixate and number of fixations) may add further to this story and may be considered in detail in future work. The fact that individuals with WS spent more time than typical attending to faces (fixated longer), yet had difficulty interpreting the cue, links to the profile of social skills associated with the disorder. Exploring individual variability of scan paths and behavioral performance in more detail is clearly warranted to consider within-syndrome variations and the heterogeneity of social skills and behaviors [42]. Indeed, exploring impacts of other behaviors associated with the disorders, such as anxiety or general social functioning, will be particularly informative in future studies. One limitation of the current study is that with the sample size used here it was not possible to take that next step and explore further the variability of gaze behavior within the WS group, linking to any subsequent differences in socio-cognitive ability or everyday skills. This remains a challenge for future research and provides an impetus for the inclusion of larger sample sizes and explorations of other social behaviors and cognitive capacities within the same individuals. Recent research from our laboratory suggests that there is large variability of social behaviors between individuals with WS that relates to inhibition abilities and which supports a frontal lobe theory of social skills in this disorder [51] – see also work on disengaging attention and shifting or controlling attention in WS [52,53].

One issue that should be noted in the current study is that images appeared on screen for a limited period and it is possible that with more time participants in both neuro-developmental disorder groups would have scanned the images differently, and perhaps even been able to process the cognitive demands of the task. With more time, therefore, participants with WS and autism may have shown a different pattern of gaze shift, but further research is required here. Task timing is important to allow participants developing atypically to attend to, process and respond to stimuli. Interestingly, Freeth and colleagues have reported atypicalities of the timing of gaze behavior (specifically attention to faces and the following of an actor’s gaze cues) in much higher functioning individuals on the autism spectrum [30]. The issue of stimuli presentation time is therefore clearly important throughout the autism spectrum. With the limitation that the current study relied on the CARS for confirmation of diagnosis (and not more detailed information, such as use of the Autism Diagnostic Observation Schedule), it was not possible to apply further examination of the effect of level of functioning on the results. The timed nature of stimulus presentation may also have affected the gaze behavior of individuals with WS who, due to their general level of mild-moderate intellectual difficulties, may utilize slower cognitive and attentional processes. Further research with different viewing times is therefore required to follow-up these preliminary suggestions. Having said this, however, it is important to bear in mind that, in real life, gaze cues indicating the target of a person’s attention are most often fleeting. Utilizing a brief presentation time therefore captures a more ecologically valid representation of this behavior in WS and autism.

One should also note that participants saw each scene twice in the two conditions (in the same order). The cognitive judgment was made the second time that the participants had attended to the stimuli, and further work may benefit from an exploration of the effect of repeat exposure on attention allocation in both typical and atypical development.

Furthermore, related to general aspects of atypical gaze and fixation in individuals with developmental disorders and the nature of the stimuli used here, it was important that the actors were embedded in scenes with items to follow for with gaze direction. However, a knock-on effect of using complex social scenes of this nature is that the areas for some of the regions of interest will by necessity be small. For example, the average eye AOI is 6.4% (minimum 5%, maximum 9%) of the height of the picture and 10.1% (minimum 8.6%, maximum 12.8%) of the width. At a viewing distance of 60 cm, this works out at almost exactly 2×1° (2.06×1.05°). However, to enlarge the face region (and consequently the eye region) within images such as this would mean that it was necessary to remove some of the complex background and have a close-up image of a face, which may not then allow an ecological insight into social scene viewing (and the related complexities of a scene). Indeed, some individuals may use visually larger information in scenes of this nature such as a full face/head to cue their gaze direction, as opposed to limiting their gaze to a smaller eye region. Systematically exploring gaze cueing when eye and head regions are congruent versus incongruent may be interesting in future research.

One interesting aspect that would have added to the analysis presented here would have been the opportunity to explore gaze behaviors for each trial independently based upon task performance (for example, whether the participant got the answer correct or incorrect). Unfortunately, due to the way the verbal response was recorded and the randomization of trials through the TobiiStudio eye-tracking program it is not possible to extract that information per trial. However, in the future it would be particularly informative to separate gaze behavior for correct and incorrect responses.

Further manipulations may also explore in more detail the impact of task instruction on gaze allocation (which can be seen as a byproduct of the current study across conditions). Indeed, the current study indicates within-group changes in the proportion of gaze to the AOIs as a function of instruction and reveals interesting issues for the autism group. For example, it is clear that the autism group realized they needed to look more at the actor’s face to answer the question (also indicating that they understood what was required in the study; for example, an understanding of task instruction) but they did not then also manage to shift their gaze to the correct target item. This provides an interesting issue that warrants further exploration.

## Conclusions

The current study provides a cross-syndrome comparison, involving individuals with WS and autism, to explore atypicalities of attention allocation and the interpretation of social cues. Although WS and autism are associated with very different social profiles, the current study indicates atypicalities in the interpretation of socio-cognitive cues in both groups. Importantly, however, these observable atypicalities are associated with very different underlying social attention atypicalities. We see syndrome-specific signatures of atypical attention allocation. Here, eye tracking has provided an excellent method to explore the group differences and how these go beyond basic observable task outcome measures. We therefore highlight the usefulness of paradigms that are able to reveal social perceptual and social cognitive atypicalities, which capture what the participants spontaneously do (and are therefore ecologically valid) and what they are capable of doing when instructed. Research of this nature therefore paves the way for using this information to inform interventions; that is, honing in on particular face skills/social attention skills necessary for social competence, and exploring the perceptual and cognitive elements. With this in mind it is possible for further research to unpick the fundamentals of social skills and behavior in typical and atypical development.

## Consent

Written informed consent was obtained from the parents of all participants for publication of this report and any accompanying images.

## Abbreviations

ANOVA: Analysis of variance; AOI: Area of interest; CARS: Childhood Autism Rating Scale; TD: Typically developing; WS: Williams syndrome.

## Competing interests

The authors declare that they have no competing interests.

## Authors’ contributions

DMR conceived the study, collected data and wrote the manuscript. PJBH helped with the study design, wrote programs, analyzed the data and helped write the manuscript. NJ helped with data extraction and analysis and manuscript preparation. MH helped with conceptualization of the study and with manuscript preparation and analysis. All authors read and approved the final manuscript.

## Supplementary Material

Additional file 1 Figures presenting all the images shown and the average gaze hotspots of participants during cued viewing. Click here for file

Additional file 2 **Figures and brief analysis of first fixation data**. Click here for file

Additional file 3 A video showing average gaze hotspots for individuals with autism during cued viewing. Click here for file

Additional file 4 A video showing average gaze hotspots for individualswith WS during cued viewing. Click here for file

Additional file 5 A video showing average gaze hotspots for TD individuals during cued viewing. Click here for file
