# Supplementary material for: Spontaneous and cued gaze-following in autism and Williams syndrome
Source: J Neurodev Disord. 2013 May 10;5(1):13. doi: 10.1186/1866-1955-5-13 (PMC3766200; doi:10.1186/1866-1955-5-13)
Supplement: Additional file 1 — Figures presenting all the images shown and the average gaze hotspots of participants during cued viewing. [file 1866-1955-5-13-S1.pdf]

Riby, Hancock, Jones & Hanley

In following images, top left is the image shown, with target AOI in red, plausible targets (target2) in blue and implausible ones (target3) in green. Top right image is average gaze hotspots during cued viewing for all the TD participants, bottom left participants with Autism, bottom right with WS.

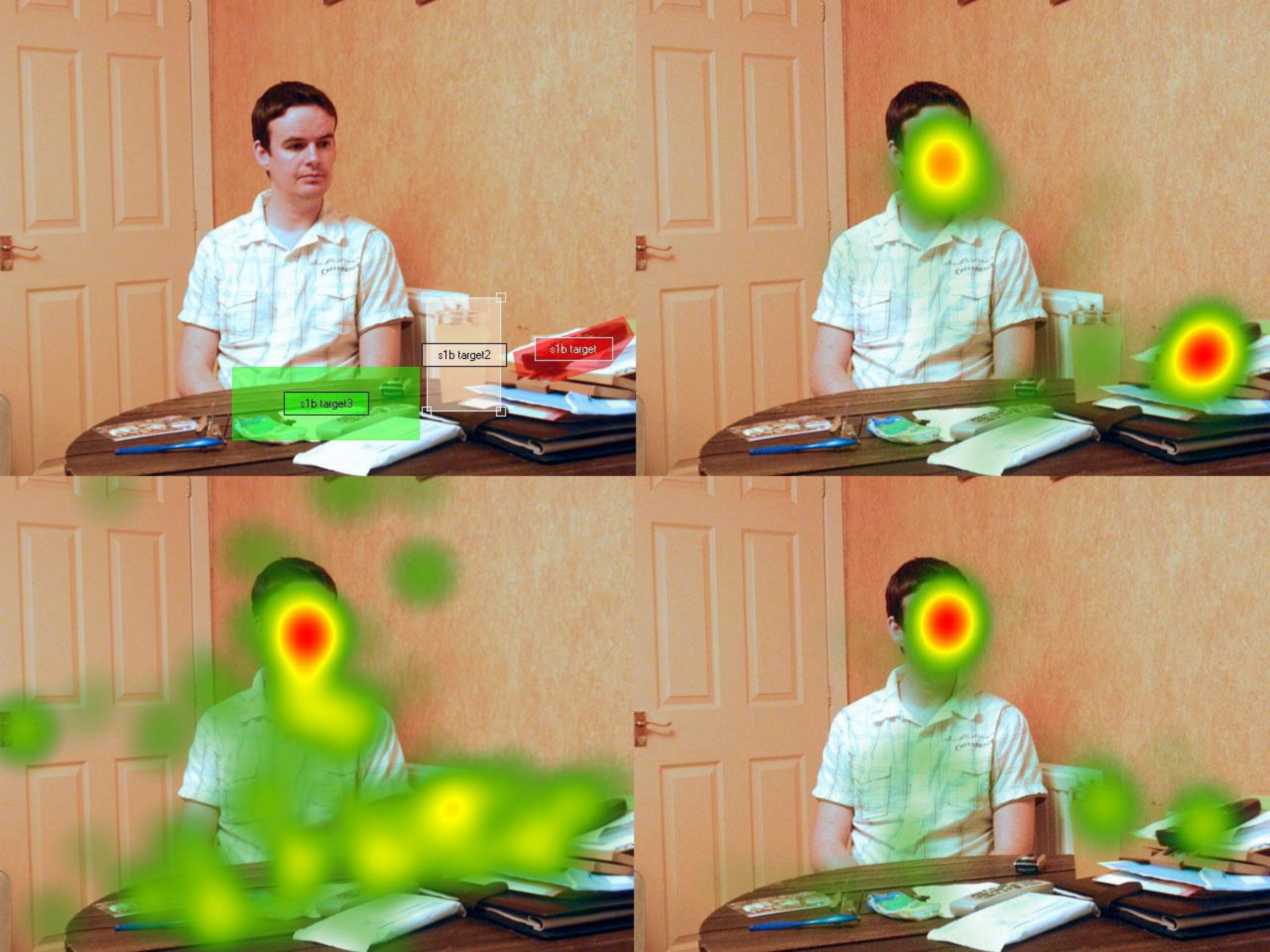
s1b target2
s1b target
s1b target3

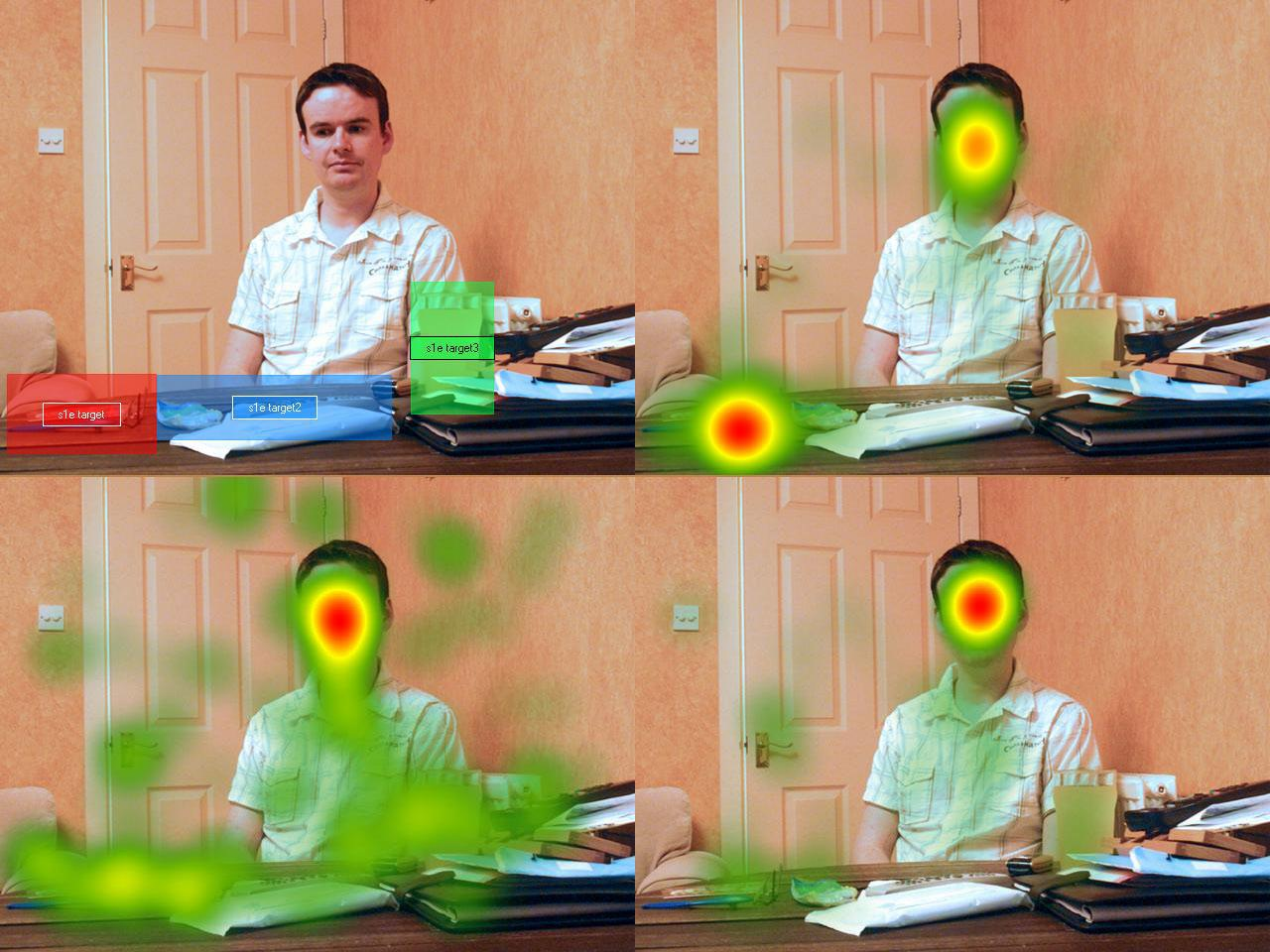
s1e target
s1e target2
s1e target3

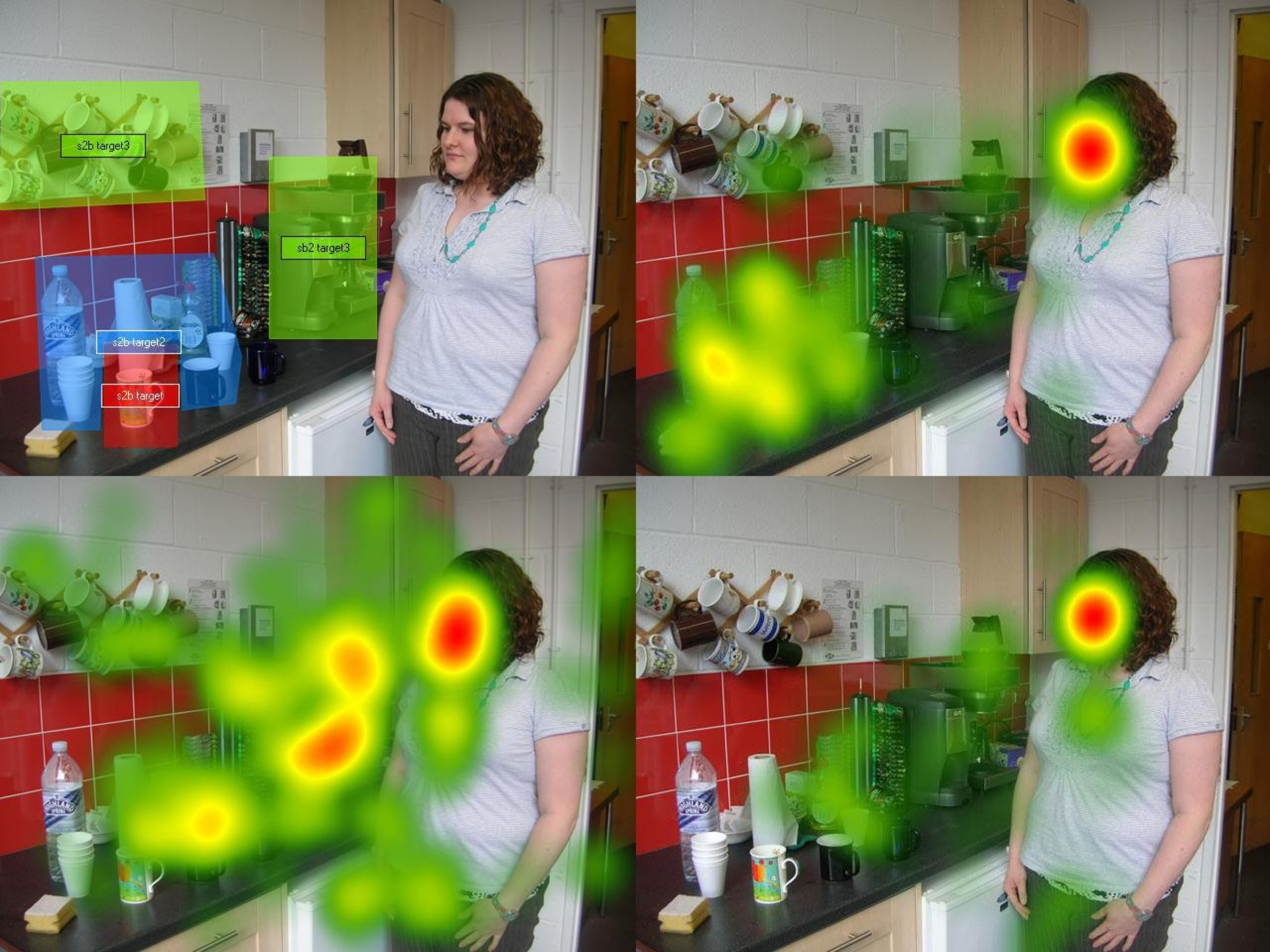
s2b target3
sb2 target3
s2b target2
s2b target
HIGHLAND SPRING

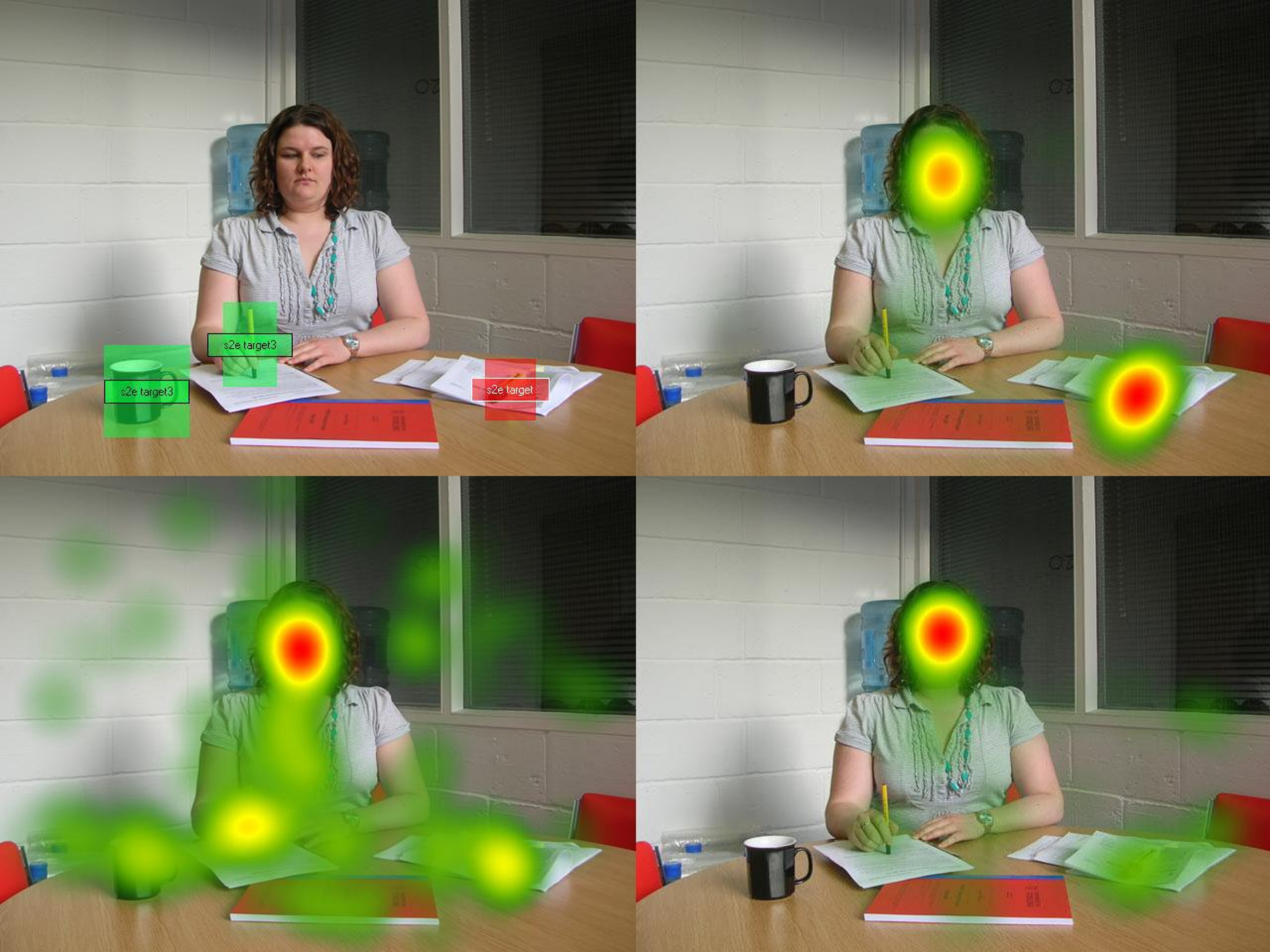
s2e target3
s2e target3
s2e target

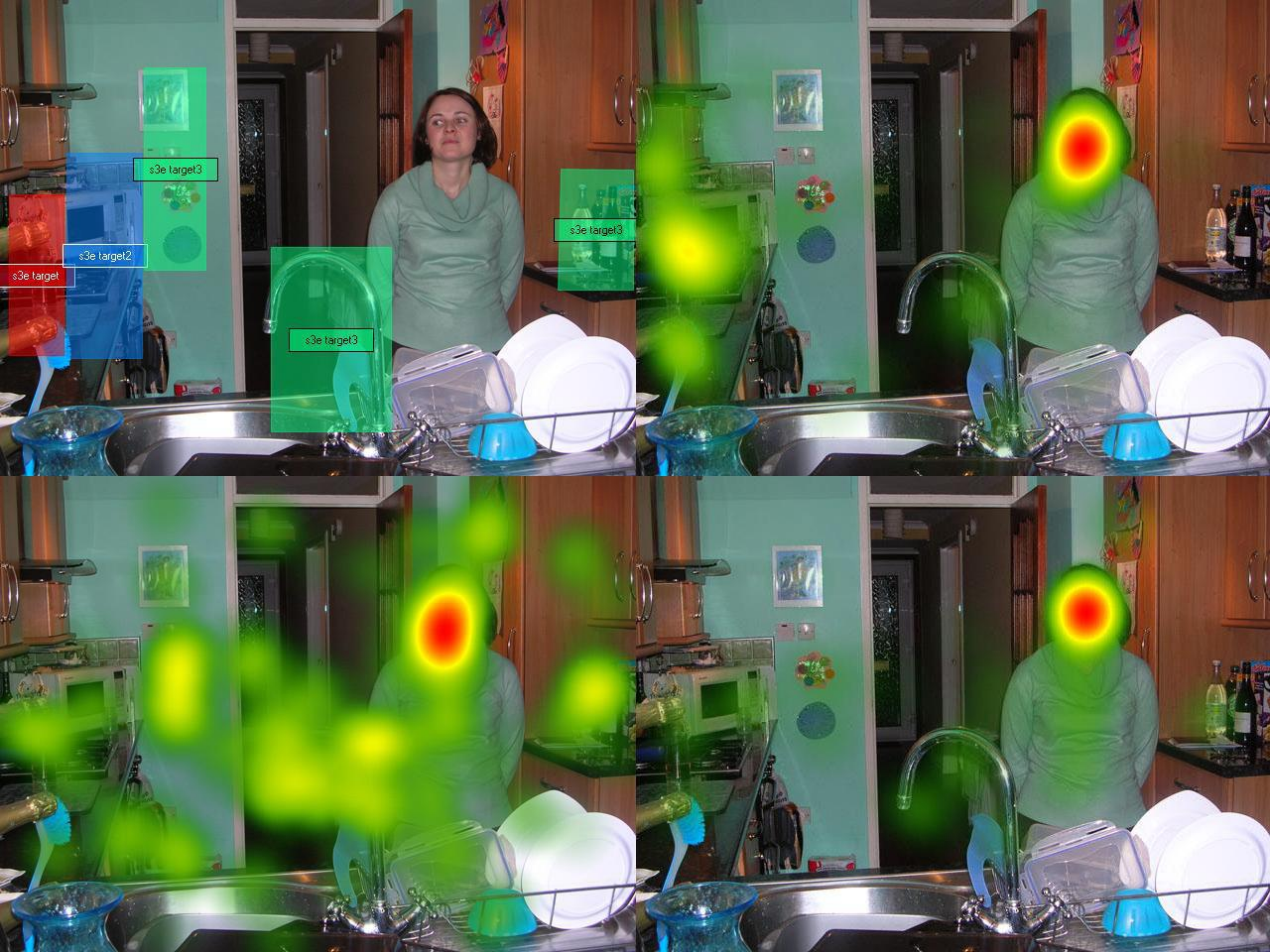
s3e target3
s3e target2
s3e target
s3e target3
s3e target3

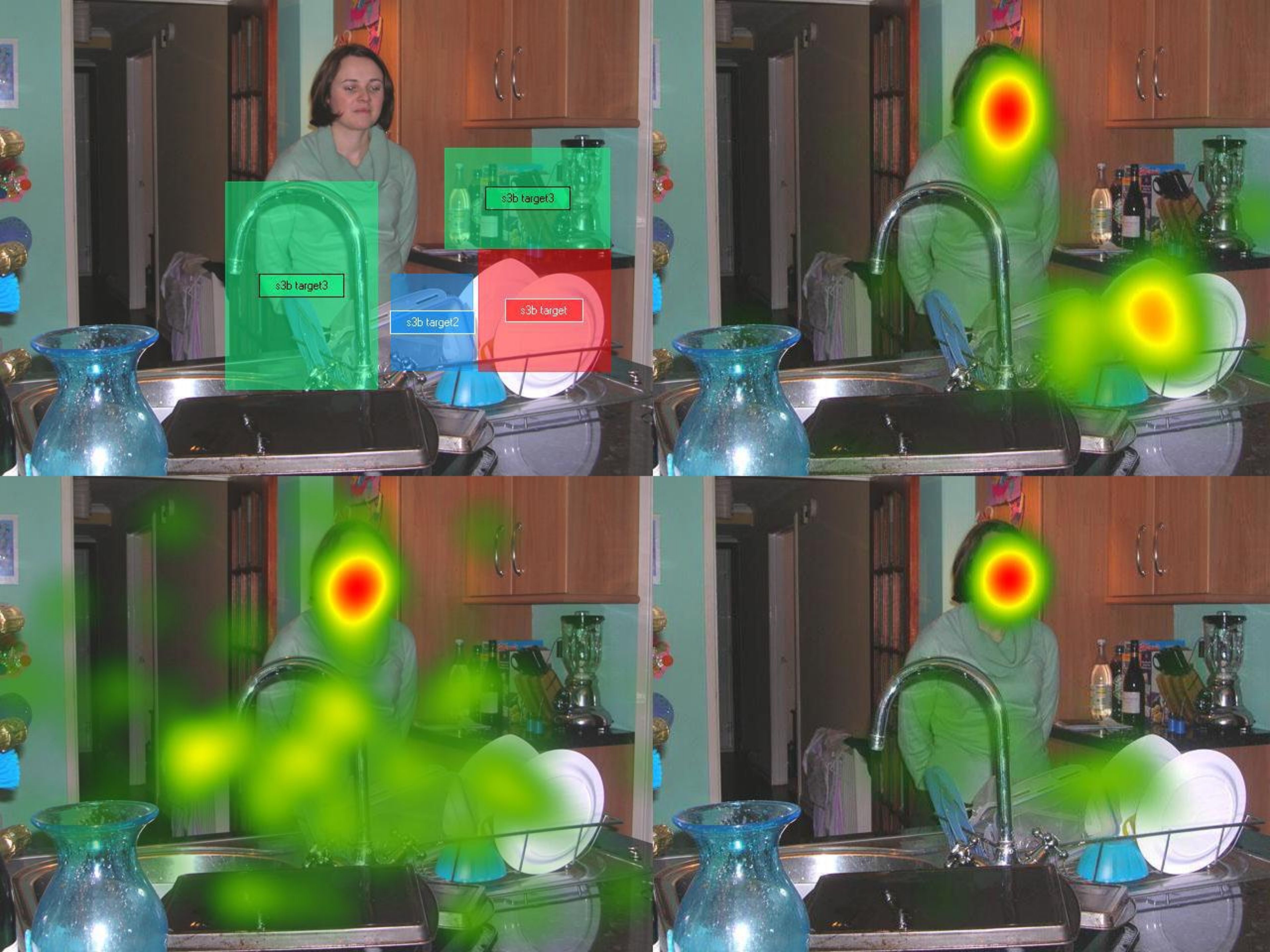
s3b target3
s3b target3
s3b target2
s3b target

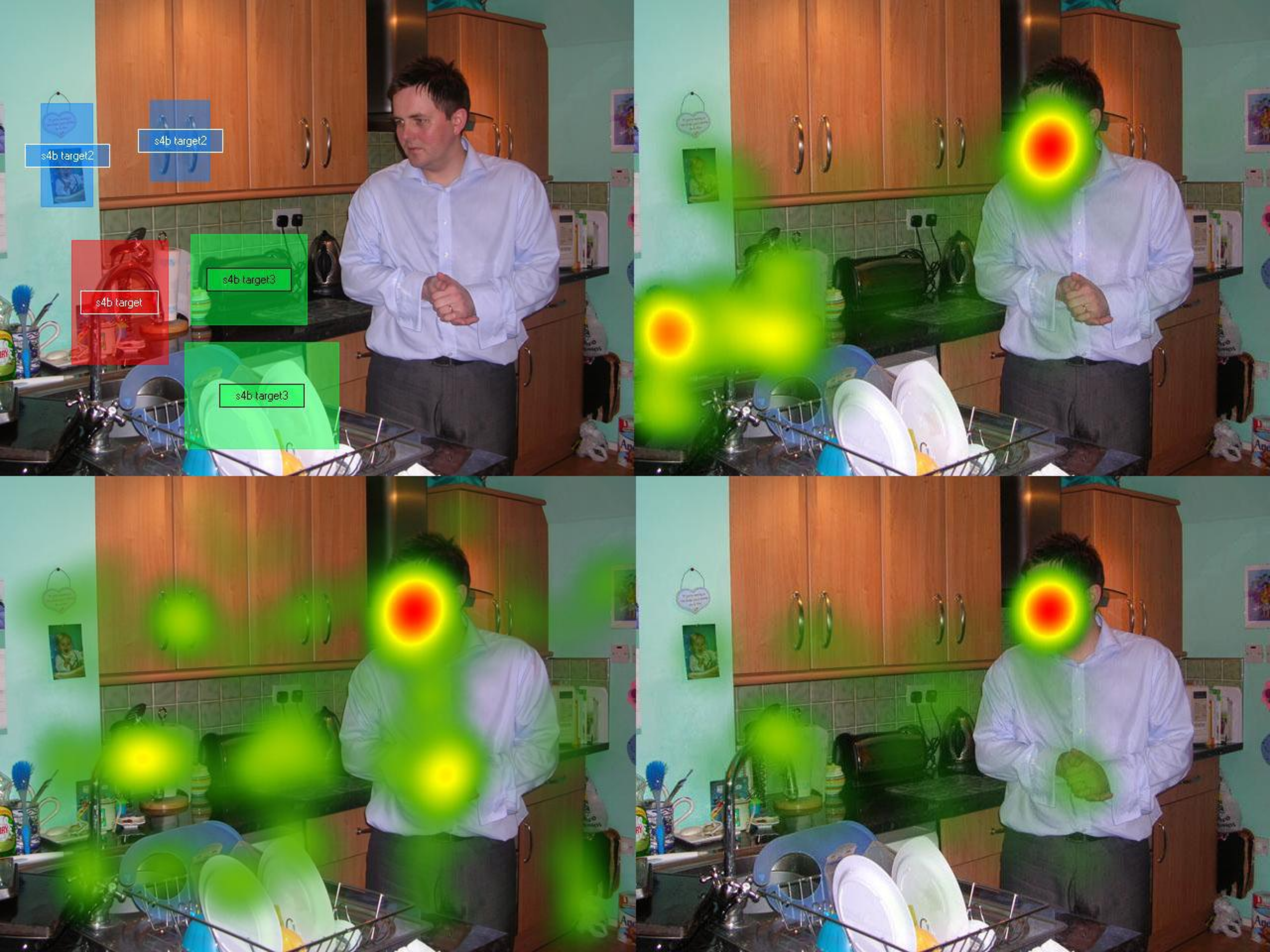
s4b target2
s4b target2
s4b target
s4b target3
s4b target3

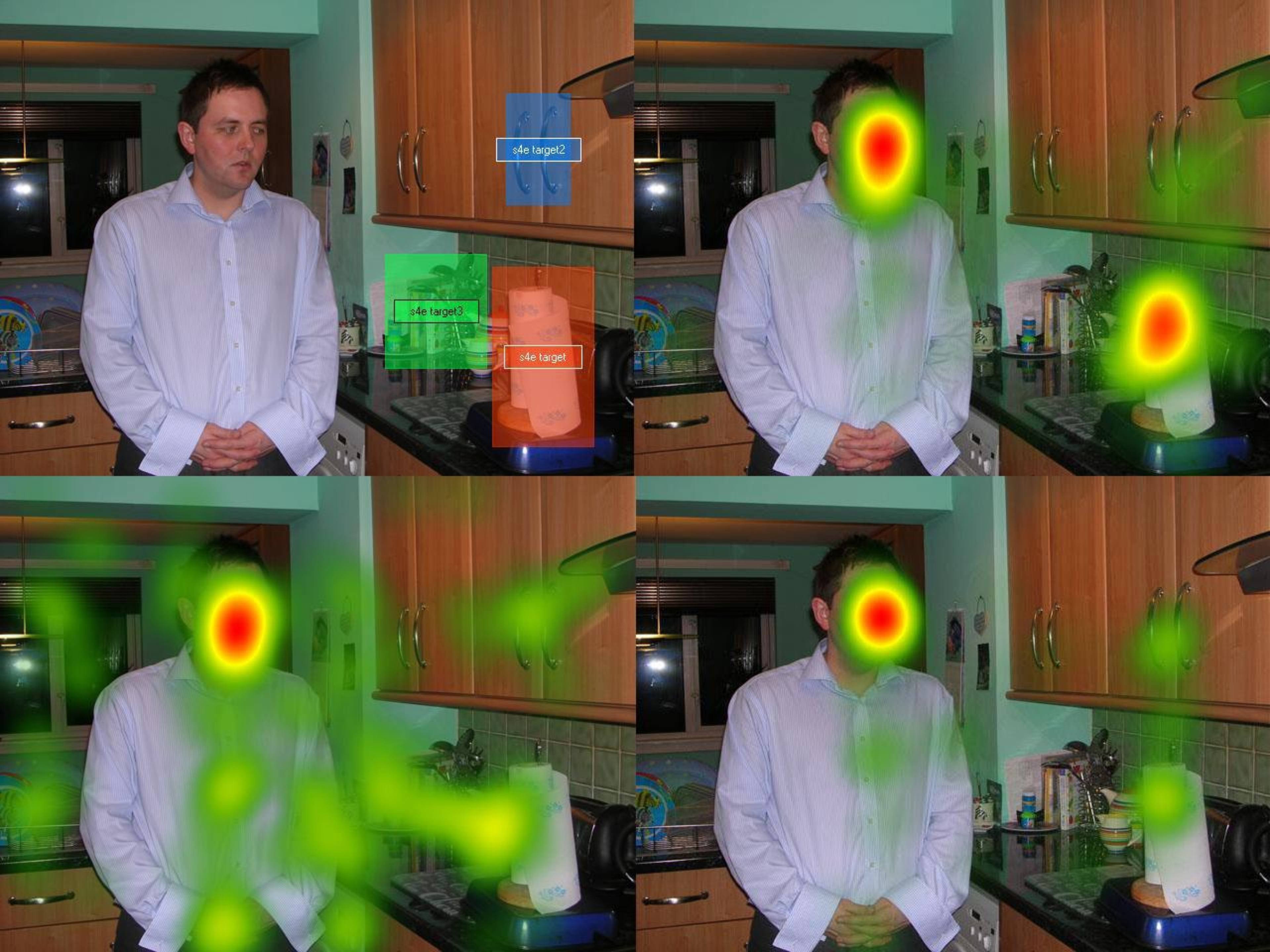
s4e target2
s4e target3
s4e target

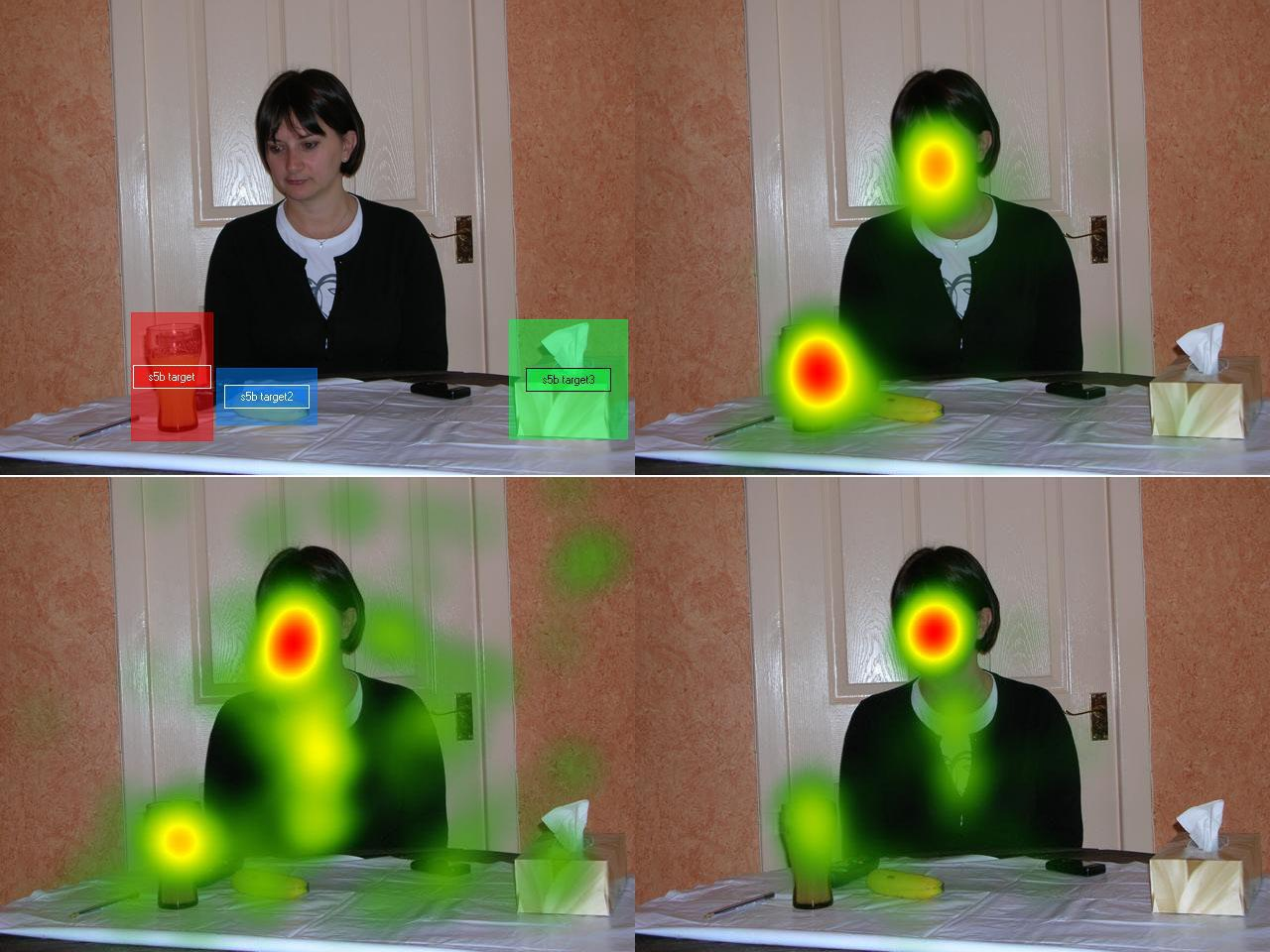
s5b target
s5b target2
s5b target3

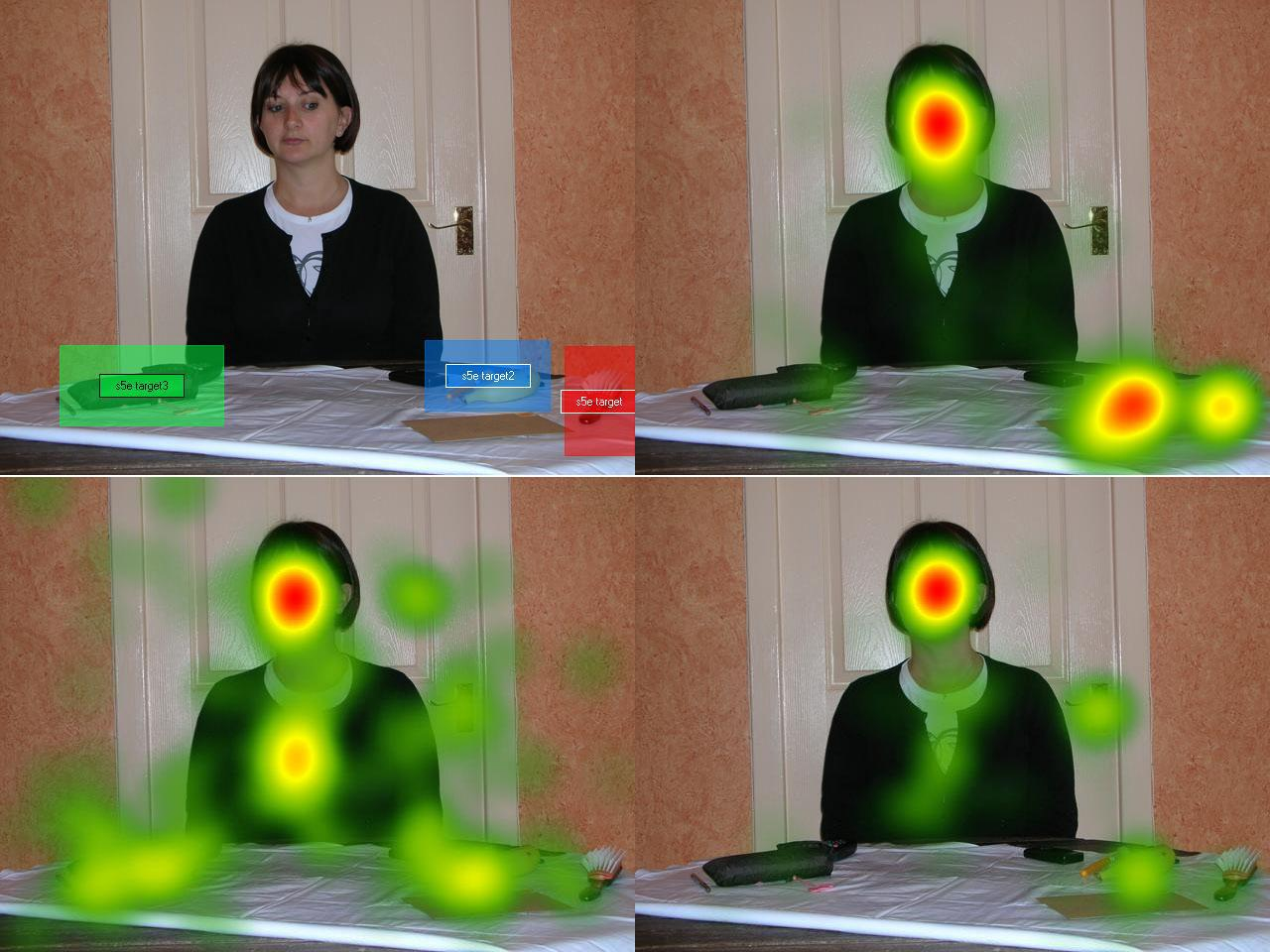
s5e target3
s5e target2
s5e target

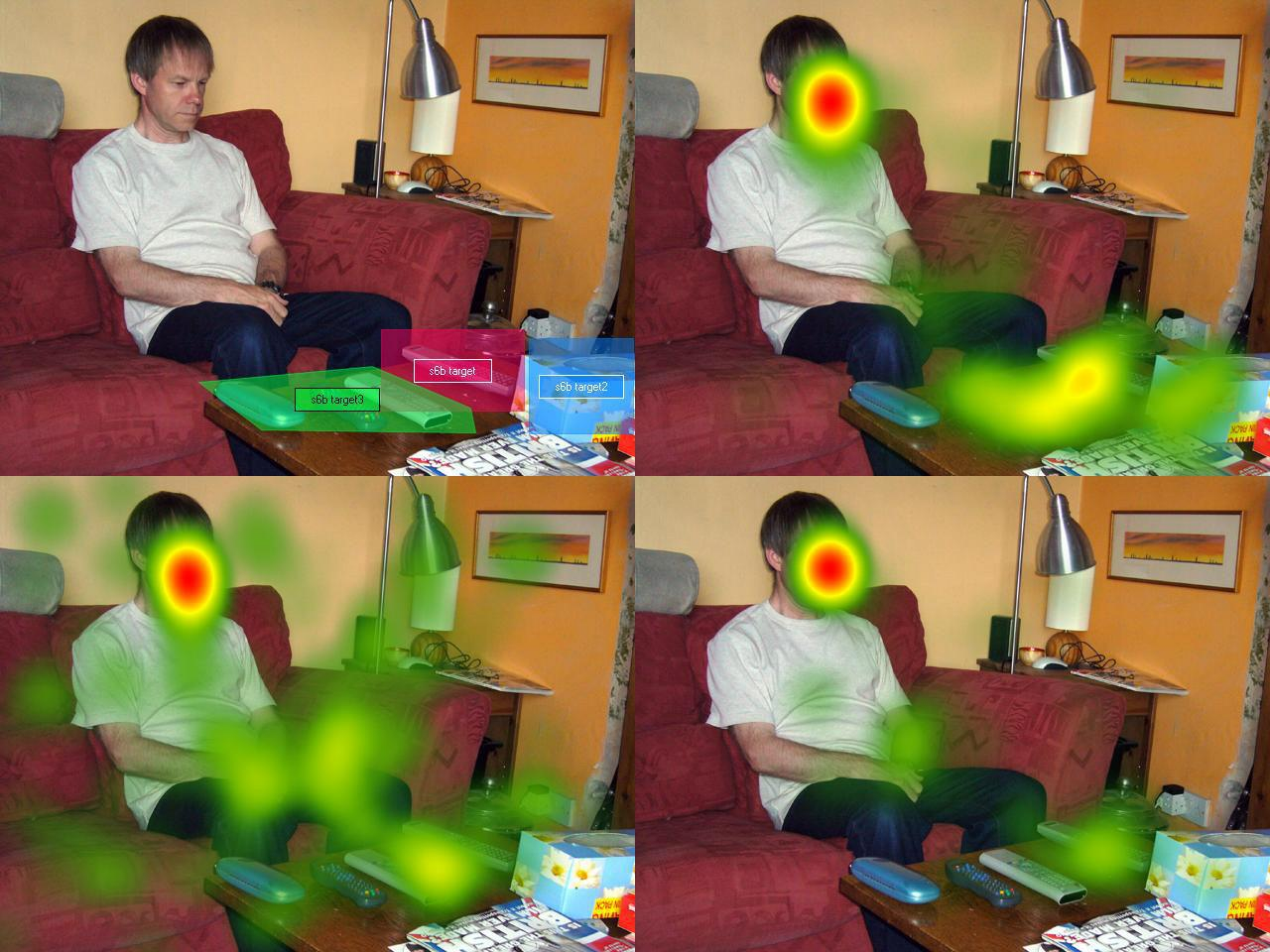
s6b target
s6b target2
s6b target3

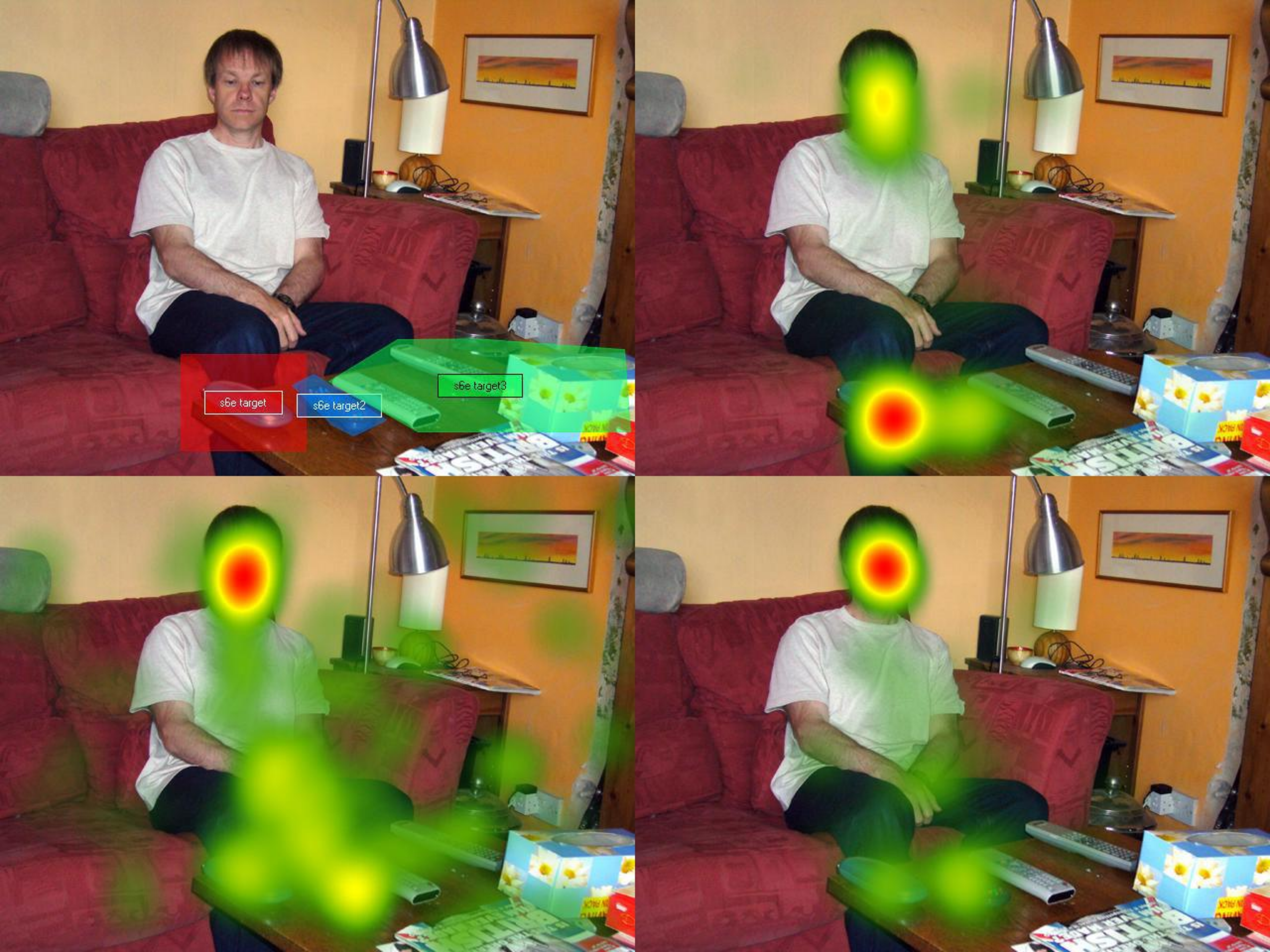
s6e target
s6e target2
s6e target3

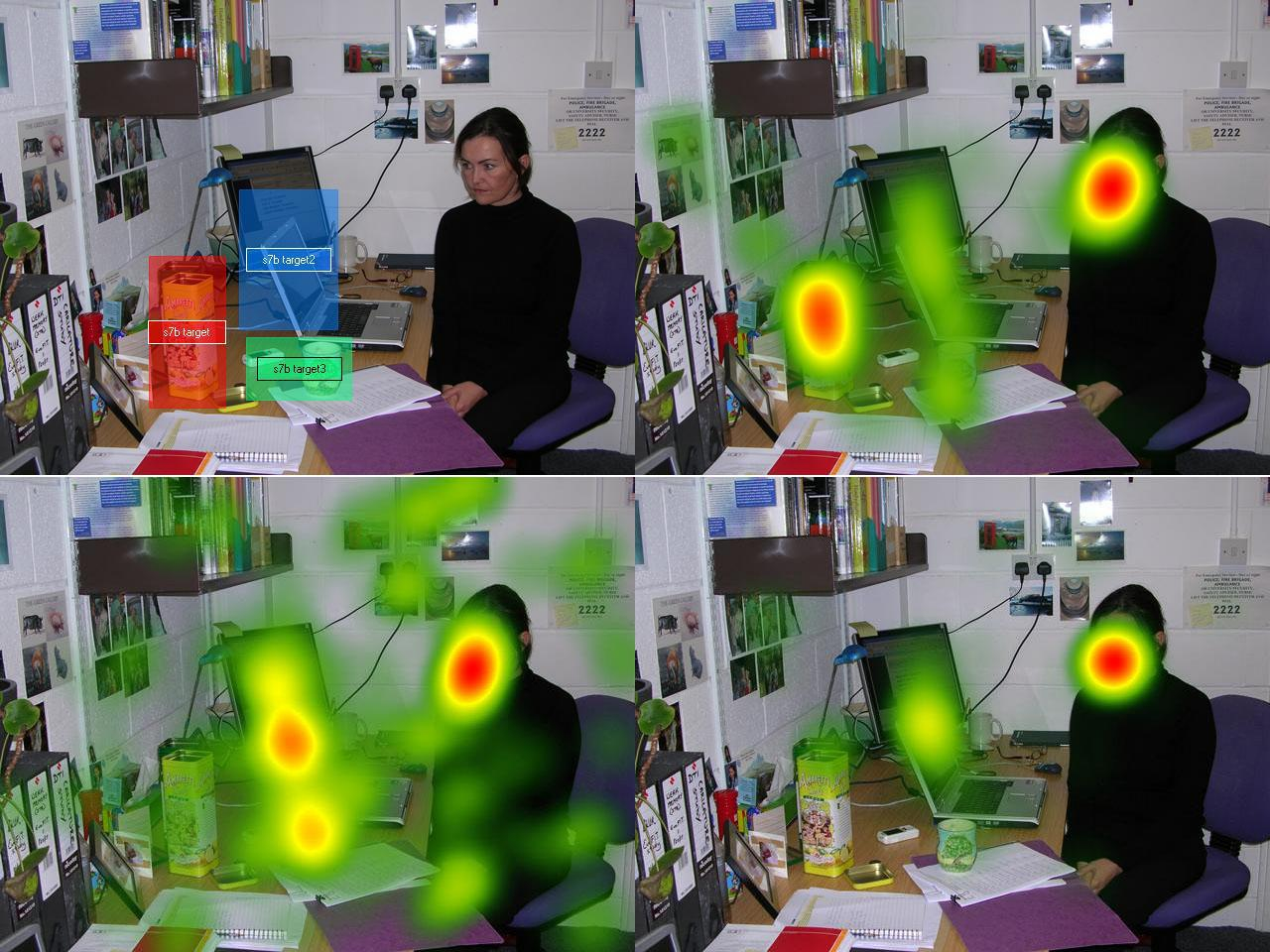
s7b target2
s7b target
s7b target3

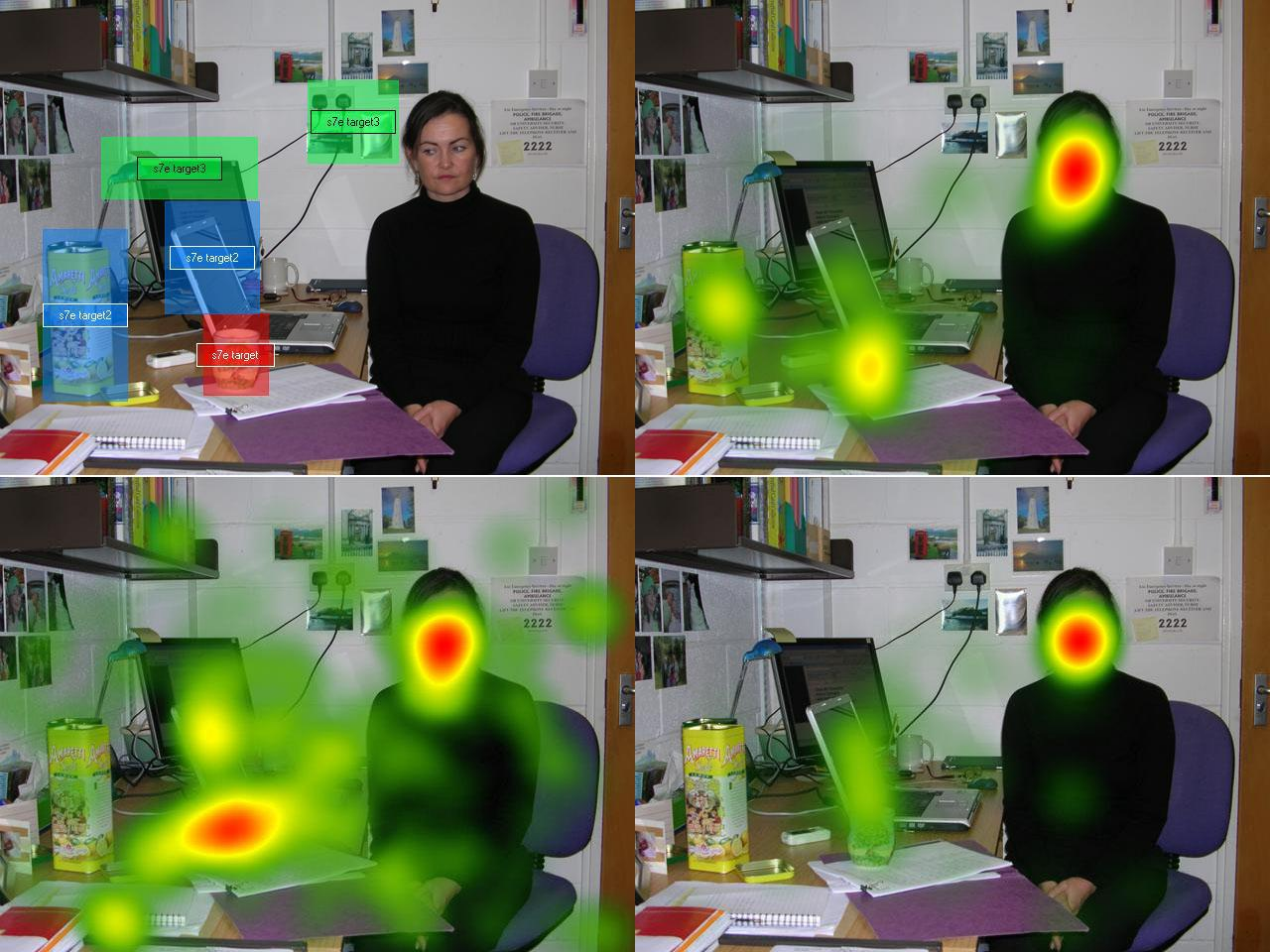
s7e target3
s7e target3
s7e target2
s7e target2
s7e target
2222
