# Supplementary material for: Spontaneous and cued gaze-following in autism and Williams syndrome
Source: J Neurodev Disord. 2013 May 10;5(1):13. doi: 10.1186/1866-1955-5-13 (PMC3766200; doi:10.1186/1866-1955-5-13)
Supplement: Additional file 2 — Figures and brief analysis of first fixation data. [file 1866-1955-5-13-S2.docx]

We calculated the average time to first fixation to each AOI for each group in each viweing condition; results are shown below.

Figure 1 Average time to first fixation in seconds for ASD (top) and WS (bottom) and their TD matches, for spontaneous and cued picture viewing

We report the summary conclusions from statistical analysis. ASD: performed same 2x2x5 ANOVA as for duration: three way interaction significant, so repeat the four 2x5 ANOVAS and again, the 2 way interactions are significant. Independent t-tests show that in free viewing, ASD take longer than TD to hit face and eyes; in cued viewing, they take longer to hit face, eyes, and correct target. Paired t-tests show that ASD take longer to hit the correct target when cued, while the TD matches take less time to hit correct target. No other pairs differ significantly.

WS: 2x2x5 three way interaction is significant, as are 2x5 2-way interactions. Independent t-tests show that in free viewing, WS take *longer* than the TD group to reach the face but less time to reach all three target types. In cued viewing, WS take *longer* to reach the correct target, no other differences significant. Paired t-tests show that the WS group take less time to fixate on face and eyes when cued, but *more* time to correct and plausible targets, no change to implausible ones. TD matches take less time to fixate on eyes and correct target when cued; no other AOIs significant.
